# Predicting cytogenetic risk in multiple myeloma using conventional whole-body MRI, spinal dynamic contrast-enhanced MRI, and spinal diffusion-weighted imaging

**DOI:** 10.1186/s13244-024-01672-1

**Published:** 2024-04-10

**Authors:** Thomas Van Den Berghe, Bert Verberckmoes, Nicolas Kint, Steven Wallaert, Nicolas De Vos, Chloé Algoet, Maxim Behaeghe, Julie Dutoit, Nadine Van Roy, Philip Vlummens, Amélie Dendooven, Jo Van Dorpe, Fritz Offner, Koenraad Verstraete

**Affiliations:** 1 Department of Radiology and Medical Imaging, Ghent University Hospital, Building -1K12, Corneel Heymanslaan 10, Ghent, B-9000 Belgium; 2 Department of Clinical Hematology, Ghent University Hospital, Corneel Heymanslaan 10, Ghent, B-9000 Belgium; 3 Department of Biostatistics, Ghent University Hospital, Corneel Heymanslaan 10, Ghent, B-9000 Belgium; 4 Center for Medical Genetics, Ghent University Hospital, Corneel Heymanslaan 10, Ghent, B-9000 Belgium; 5 Department of Pathology, Ghent University Hospital, Corneel Heymanslaan 10, Ghent, B-9000 Belgium

**Keywords:** Diffusion magnetic resonance imaging, Genetics, Magnetic resonance imaging, Multiparametric magnetic resonance imaging, Multiple myeloma

## Abstract

**Objectives:**

Cytogenetic abnormalities are predictors of poor prognosis in multiple myeloma (MM). This paper aims to build and validate a multiparametric conventional and functional whole-body MRI-based prediction model for cytogenetic risk classification in newly diagnosed MM.

**Methods:**

Patients with newly diagnosed MM who underwent multiparametric conventional whole-body MRI, spinal dynamic contrast-enhanced (DCE-)MRI, spinal diffusion-weighted MRI (DWI) and had genetic analysis were retrospectively included (2011–2020/Ghent University Hospital/Belgium). Patients were stratified into standard versus intermediate/high cytogenetic risk groups. After segmentation, 303 MRI features were extracted. Univariate and model-based methods were evaluated for feature and model selection. Testing was performed using receiver operating characteristic (ROC) and precision-recall curves. Models comparing the performance for genetic risk classification of the entire MRI protocol and of all MRI sequences separately were evaluated, including all features. Four final models, including only the top three most predictive features, were evaluated.

**Results:**

Thirty-one patients were enrolled (mean age 66 ± 7 years, 15 men, 13 intermediate-/high-risk genetics). None of the univariate models and none of the models with all features included achieved good performance. The best performing model with only the three most predictive features and including all MRI sequences reached a ROC-area-under-the-curve of 0.80 and precision-recall-area-under-the-curve of 0.79. The highest statistical performance was reached when all three MRI sequences were combined (conventional whole-body MRI + DCE-MRI + DWI). Conventional MRI always outperformed the other sequences. DCE-MRI always outperformed DWI, except for specificity.

**Conclusions:**

A multiparametric MRI-based model has a better performance in the noninvasive prediction of high-risk cytogenetics in newly diagnosed MM than conventional MRI alone.

**Critical relevance statement:**

An elaborate multiparametric MRI-based model performs better than conventional MRI alone for the noninvasive prediction of high-risk cytogenetics in newly diagnosed multiple myeloma; this opens opportunities to assess genetic heterogeneity thus overcoming sampling bias.

**Key points:**

• Standard genetic techniques in multiple myeloma patients suffer from sampling bias due to tumoral heterogeneity.

• Multiparametric MRI noninvasively predicts genetic risk in multiple myeloma.

• Combined conventional anatomical MRI, DCE-MRI, and DWI had the highest statistical performance to predict genetic risk.

• Conventional MRI alone always outperformed DCE-MRI and DWI separately to predict genetic risk. DCE-MRI alone always outperformed DWI separately, except for the parameter specificity to predict genetic risk.

• This multiparametric MRI-based genetic risk prediction model opens opportunities to noninvasively assess genetic heterogeneity thereby overcoming sampling bias in predicting genetic risk in multiple myeloma.

**Graphical Abstract:**

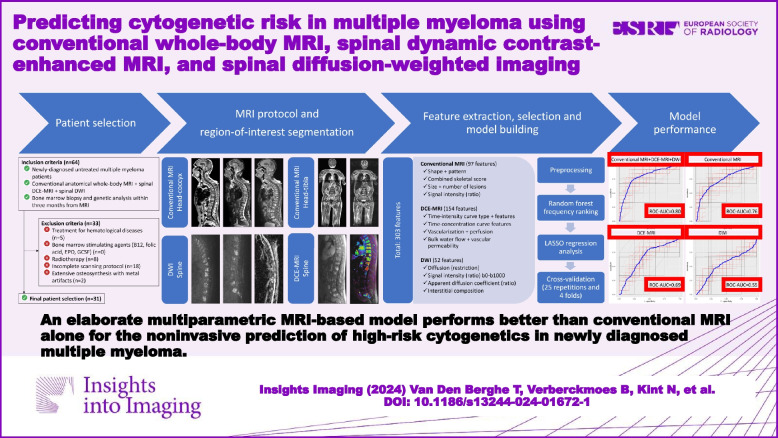

**Supplementary Information:**

The online version contains supplementary material available at 10.1186/s13244-024-01672-1.

## Introduction

Multiple myeloma (MM) consists of a proliferation of malignant plasma cells in the bone marrow (BM) with an overproduction of monoclonal proteins (M-protein) [1]. Symptomatic MM is characterized by end-organ damage and dysfunction, as specified by the SLiM-CRAB criteria [2, 3]. It accounts for 1% of neoplastic disorders and 10% of hematological cancers and is the second most common hematological malignancy. It is responsible for 5–20% of deaths from hematological malignancies and 2% of all cancer deaths [4–7].

MM is a collection of cytogenetically distinct disorders. Approximately 40% is characterized by odd chromosome 3, 5, 7, 9, 11, 15, 19 trisomies (trisomic/hyperdiploid MM), while the rest predominantly has a translocation of the immunoglobulin heavy chain (IgH) locus with proto-oncogenes as partners (chromosome 14q32; IgH-translocated MM) [8, 9]. Trisomies and IgH translocations are primary cytogenetic abnormalities (CA) at disease initiation. Secondary CAs arise, including gain (1q21)/del(1p22/32)/del(17p13)/del(13/13q14)/RAS-mutations/MYC-translocations, leading to tumor progression [10]. CAs influence disease course, response to therapy, and progression [5–7, 11–14]. Median overall survival (OS) is 6–7 years, but with important inter-patient variability, ranging from < 1 year to > 10 years. Adverse risk factors depend on host factors including tumor burden, extramedullary disease, CAs, and therapy response [9]. Patients with standard-risk CAs have a median OS of 7–10 years while patients with intermediate-/high-risk CAs have a median OS of 2–5 years, shorter time-to-relapse, inferior therapy response, more extramedullary disease, and more organ failure at diagnosis [8, 9, 15–18]. Clinical risk models included high-risk CAs such as t(4;14)(p16;q32)/t(14;16)(q32;q23)/t(14;20)(q32;q11)/del(17p13)/non-hyperdiploidy/gain and amplification (1q21)/del(1p22/32)/del(13/13q14) [International Myeloma Working Group (IMWG), International Staging System Second Revision (R2-ISS), (updated) Mayo Clinic Risk Stratification for MM (mSMART)] [5–7, 19–26].

Due to the importance of CAs in MM, the IMWG defined minimal recommendations for genetic analysis for identification of numerical abnormalities, translocations and other CAs, including conventional karyotyping and interphase fluorescence in situ hybridization (iFISH) [20, 23, 24].

Radiogenomics is used for noninvasive genotyping and risk stratification by using clinical images to identify predictive imaging biomarkers. It captures inter- and intra-tumoral genetical heterogeneity, thereby reducing the potential limitations of biopsy sampling error [20, 27]. Conventional anatomical MRI is adopted by the IMWG as a routine imaging modality in MM and has the highest sensitivity and specificity in detecting BM infiltration [6, 11, 28–35]. Dynamic contrast-enhanced (DCE-)MRI and diffusion-weighted imaging (DWI) hold additional value in assessing BM infiltration and physiology and allow for the assessment of vascularization/perfusion/bulk water flow/capillary permeability (DCE-MRI) and water content/diffusion capacity/interstitial composition (DWI) [36–40]. Previous studies investigated the potential of MRI to predict cytogenetic risk in MM patients on specific MRI sequences and with various techniques. None of them assessed the potential of extensive qualitative/(semi-)quantitative whole-body multiparametric MRI as used in the current study. Radiogenomics using multiparametric MRI has the potential to noninvasively stratify genetic risk and to facilitate precision oncology.

The goal of this study is to build and test an extensive multiparametric combined conventional anatomical and functional MRI-based model to predict high-risk CAs in newly diagnosed MM patients to be used as a first study.

## Methods

Ethics committee approval [EC2019-1267(BC-06060)/1268(BC-06063)] and written informed consent were obtained for retrospective analysis by the Institutional Review Board (Ghent University Hospital, Belgium).

### Study group

**Fig. 1:**
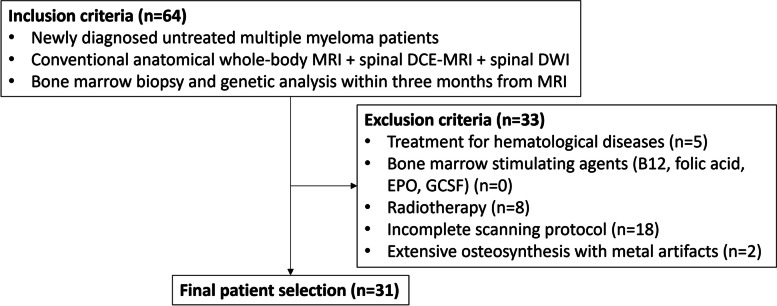
Patient flowchart with inclusion criteria and initial retrieval, exclusion criteria and final patient selection. *B12* vitamin B12, *DCE* dynamic contrast-enhanced, *DWI* diffusion-weighted imaging, *EPO* erythropoietin, *GCSF* granulocyte colony-stimulating factor, *IMWG* international myeloma working group, *MRI* magnetic resonance imaging, *n* number

Retrospective consecutive inclusion, exclusion, and final patient selection at the Ghent University Hospital (Belgium, 2011–2020) and patient characteristics are summarized in Fig. 1 and Table 1 [41]. All patients presenting with newly diagnosed MM that were finally included in the study were diagnosed by a tertiary hospital hematologist according to the IMWG criteria (laboratory/clinical/histopathological/imaging information) and were referred to the radiology department for an extensive whole-body MRI examination (see section “Imaging”) [6].

**Table 1:** Patient characteristics and clinical information of the entire patient population, the intermediate-/high-, and the standard-risk cytogenetic group [26, 40, 41]

	**All patients** (***n* = 31, 100%)**	**Intermediate-/high-risk** (***n* = 13, 42%)**	**Standard-risk** (***n* = 18, 58%)**
**Patient characteristics**			
Gender (male–female)	15 (48%)–16 (52%)	6 (46%)–7 (54%)	9 (50%)–9 (50%)
Genetics-MRI (months)	-0.3 ± 1.5 (-2.5–3.0)	0.0 ± 1.5 (-1.8–2.6)	-0.6 ± 1.4 (-2.2–3.0)
Death	6 (19%)	3 (23%)	3 (17%)
Age at diagnosis (years)	66.4 ± 7.4	68.0 ± 6.4	65.3 ± 8.1
BMI (kg/m^2^)	26.3 ± 4.5	26.2 ± 4.9	26.4 ± 4.3
Two-year overall survival	97%	92%	100%
Three-year overall survival	94%	92%	94%
**Laboratory results**			
MM subdiagnosis	7 IgAλ (23%)−4 IgAκ (13%)−5 IgGλ (16%)−2 IgGκ (39%)−1 LCλ (3%)−1 LCκ (3%)−1 IgMκ (3%)	2 IgAλ (15%)−2 IgAκ (15%)−2 IgGλ (15%)−6 IgGκ (47%)−1 LCλ (8%)−0 LCκ (0%)−0 IgMκ (0%)	5 IgAλ (28%)−2 IgAκ (11%)−3 IgGλ (17%)−6 IgGκ (34%)−0 LCλ (0%)−1 LCκ (5%)−1 IgMκ (5%)
BSR (mm/hour)^a^	52.5 ± 37.7	57.9 ± 40.6	49.5 ± 37.0
Hemoglobin (g/dL)^b^	12.3 ± 1.9	12.1 ± 2.1	12.5 ± 1.7
Hematocrit (%)^c^	37.1 ± 4.8	36.5 ± 5.4	37.5 ± 4.4
Anemia	16 (52%)	8 (62%)	8 (44%)
Calcium (mg/dL)^d^	9.6 ± 0.9	9.4 ± 0.6	9.7 ± 1.1
Calcium status	28 normal (90%)–2 hypo (7%)–1 hyper (3%)	12 normal (92%)–1 hypo (8%)–0 hyper (0%)	16 normal (88%)–1 hypo (6%)–1 hyper (6%)
Creatinine (mg/dL)^e^	0.9 ± 0.3	0.9 ± 0.2	1.0 ± 0.3
GFR (mL/min/1.73m^2^)^f^	72.7 ± 15.5	75.8 ± 13.4	70.4 ± 16.8
CKD^g^	6G1 (19%)–20G2 (65%)–4G3a (13%)–1G3b (3%)–0G4 (0%)–0G5 (0%)	4G1 (31%)–8G2 (61%)–1G3a (8%)–0G3b (0%)–0G4 (0%)–0G5 (0%)	2G1 (11%)–12G2 (67%)–3G3a (17%)–1G3b (5%)–0G4 (0%)–0G5 (0%)
β2-microglobulin (mg/L)^h^	2.9 ± 1.1	2.9 ± 1.3	3.0 ± 1.0
Protein absolute (g/L)^i^	77.0 ± 15.8	80.8 ± 11.9	74.3 ± 17.9
Albumin absolute (g/L)^j^	39.3 ± 4.9	39.6 ± 5.5	39.1 ± 4.5
Gamma globulins (%)^k^	25.0 ± 13.3	22.7 ± 11.8	26.6 ± 14.4
M-protein peak (g/L)	18.2 ± 11.3	18.9 ± 11.0	17.7 ± 12.1
IF global extrafraction	10 no suppression (32%)	5 no suppression (38%)	5 no suppression (28%)
	21 suppression (68%)	8 suppression (62%)	13 suppression (72%)
LDH (U/L)^l^	176.3 ± 49.4	187.8 ± 49.1	168.0 ± 49.3
CRP (mg/L)^m^	6.0 ± 12.6	7.8 ± 14.4	4.8 ± 11.4
M-protein absolute (g/L)	63.9 ± 147.9	77.3 ± 186.6	54.2 ± 117.6
M-protein relative^n^	4.9 ± 6.6	4.7 ± 6.7	5.0 ± 6.8
LC involved/uninvolved	35.2 ± 67.2	27.9 ± 35.2	40.5 ± 83.8
BJ proteins urine	18 no (58%)–9 κ (29%)–4 λ (13%)	10 no (77%)–2 κ (15%)–1 λ (8%)	8 no (44%)–7 κ (39%)–﻿3 λ (17%)
WHO myelofibrosis score^o^	22MF0 (71%)–7MF1 (23%)	9MF0 (69%)–4MF1 (31%)	13MF0 (72%)–3MF1 (17%)
	2MF2 (6%)–0MF3 (0%)	0MF2 (0%)–0MF3 (0%)	2MF2 (11%)–0MF3 (0%)
BMP plasmacytosis (%)	37.7 ± 20.6	35.7 ± 20.5	38.7 ± 21.3
**Genetics**			
Diploidy	17 diploid (55%)	6 diploid (46%)	11 diploid (61%)
	3 hypodiploid (10%)	3 hypodiploid (23%)	0 hypodiploid (0%)
	11 hyperdiploid (35%)	4 hyperdiploid (31%)	7 hyperdiploid (39%)
Gain(1q)-del(1p)-del(13q)	3 (10%)-3 (10%)-3 (10%)	3 (23%)-3 (23%)-3 (23%)	0 (0%)-0 (0%)-0 (0%)
Del(17(p13))-TP53	2 (6%)	2 (15%)	0 (0%)
t(4;14)(q16;q32)	1 (3%)	1 (8%)	0 (0%)
t(11;14)(q13;q32)	2 (6%)	0 (0%)	2 (11%)
t(14;16)(q32;q23)	0 (0%)	0 (0%)	0 (0%)
t(14;20)(q32;q11)	0 (0%)	0 (0%)	0 (0%)
t(6;14)(p21;q32)	0 (0%)	0 (0%)	0 (0%)
Trisomy odd Cx^p^	11 (35%)	5 (38%)	6 (33%)
del(6q,8p,13,11q,14q,16q)	7 (23%)	7 (54%)	0 (0%)
**Risk stratification**			
SDP^q^	18 I (58%)-13 II (42%)-0 III (0%)	5 I (38%)-8 II (62%)-0 III (0%)	13 I (72%)-5 II (28%)-0 III (0%)
R2-ISS^r^	16 I (52%)-15 II (48%)-0 III (0%)	6 I (46%)-7 II (54%)-0 III (0%)	10 I (56%)-8 II (44%)-0 III (0%)

*BJ* Bence Jones, *BMI* Body mass index, *BMP* Bone marrow puncture, *BSR* Blood sedimentation rate, *CKD* Chronic kidney disease, *CRP* C-reactive protein, *Cx* Chromosome, *del* Deletion, *dL* Deciliter, *g* Gram, *G1-5* Grade of chronic kidney disease, *GFR* Glomerular filtration rate, *IF* Immunofluorescence, *Ig* Immunoglobulin, *kg* Kilogram, *L* Liter, *LC* Light chain, *LDH* Lactate dehydrogenase, *m*^*2*^ Square meter, *MF* Myelofibrosis, *mg* Milligram, *min* Minute, *mL* Milliliter, *mm* Millimeter, *MM* Multiple myeloma, *M-protein* Monoclonal protein, *n* Number, *p* Probability, *R2-ISS* Second revision of the international staging system, *SDP* Salmon and Durie plus staging system, *t(x;y)* Chromosomal translocation (x,y), *TP53* Tumor protein P53, *WHO* World Health Organization

Normal values and stadiums: ^a^first hour 0–30; ^b^11.8–14.8; ^c^35.8–43.7; ^d^8.5–10.5; ^e^0.55–0.96; ^f^according to CKD-EPI, normal values are dependent on the stage of chronic kidney disease; ^g^based on glomerular filtration rate (G1–normal and high ≥ 90, G2–mild reduction related to normal range for a young adult 60–90, G3a–mild to moderate reduction 45–59, G3b–moderate to severe reduction 30–44, G4–severe reduction 15–29, G5–kidney failure < 15); ^h^1.09–2.53; ^i^62–81; ^j^35–52; ^k^8.7–17.7; ^l^105–250; ^m^ < 5.0; ^n^as compared to maximal normal values of immunoglobulins (IgG 7.0–16.0 g/L, IgM 0.40–2.48 g/L, IgA 0.71–3.65 g/L, light-chain kappa 6.7–22.4 mg/L, light-chain lambda 8.3–27.0 mg/L); ^o^reticulin pattern on bone marrow biopsy (MF0–scattered linear reticulin with no intersections (cross-overs) corresponding to normal bone marrow, MF1–loose network of reticulin with many intersections, especially in perivascular areas, MF2–diffuse and dense increase in reticulin with extensive intersections, occasionally with only focal bundles of collagen and/or focal osteosclerosis, MF3–diffuse and dense increase in reticulin with extensive intersections with coarse bundles of collagen, often associated with significant osteosclerosis); ^p^3,5,7,9,11,15,19; ^q^original Salmon and Durie staging system extended with number of focal lesions on whole-body MRI/PET (stadium I: 0–4, stadium II: 5–20, stadium III: > 20); ^r^original international staging system extended (second revision) with FISH/chromosomal abnormality analysis and lactate dehydrogenase analysis (stadium I: standard-risk cytogenetics and normal lactate dehydrogenase, stadium II: not stadium I or III, stadium III: high-risk cytogenetics and/or high lactate dehydrogenase)

### Clinical parameters

Relevant clinical parameters are displayed in Table 1 [40, 41]. The percentage of CD138-/CD38-/MUM1-positive monoclonal plasma cells and the pattern of myelofibrosis on BM biopsy was determined by two independent blinded pathologists (J.V.D./A.D., 33/16 years experience).

### Genetic analysis as reference standard

Iliac crest BM biopsies underwent obligatory testing with array comparative genomic hybridization (array CGH) or copy-number variation sequencing (CNV-seq, shallow whole-genome sequencing) for assessment of ploidy and non-obligatory testing with iFISH on CD138-expressing plasma cells (chromosome 14 translocations) [42–45]. Ploidy was classified as hyperdiploid (≥ 47 chromosomes), pseudodiploid (45–46 chromosomes), or hypodiploid (≤ 44 chromosomes). According to the R2-ISS, the (updated) mSMART and the IMWG guidelines, CAs were classified as intermediate-/high-risk or standard-risk (Table 2).

**Table 2:** Cytogenetic abnormalities with associated genes and frequency in multiple myeloma and differences between intermediate-/high-risk and standard-risk cytogenetics. For high-risk cytogenetic abnormalities, the presence of two high-risk factors is considered double-hit myeloma. Three or more high-risk factors is considered triple-hit myeloma [5, 7, 9, 10, 15, 19–24, 26, 46–53]

Intermediate-/high-risk cytogenetics and CAs (25%)			Standard-risk cytogenetics and CAs (75%)			
*Abnormality*	*Gene(s)*	*Frequency*	*Abnormality*	*Gene(s)*	*Frequency*	
GENERAL: non-hyperdiploid^d, h, k^			GENERAL: hyperdiploid^d^			
t(4;14)(p16;q32)^c, d, g, h, k^	FGFR3/MMSET	6–15%	t(11;14)(q13;q32)^c, d^	CCND1		15–20%
t(14;16)(q32;q23)^c, d, g, i, k^	C-MAF/CCND2	1–7%	t(6;14)(p21;q32)^c, d^	CCND3		1–5%
t(14;20)(q32;q11)^c, d, i, k^	MAFB/CCND2	1–2%	trisomy odd Cx^b, d^	NA / multiple		42–50%
del(1p22/32)^f, k^	CDKN2C/FAF1/FAM46C	20–30%				
del(17(p13))^a, f, g, i, k^	TP53	5–11%				
gain(3 copies)/amp(≥ 4 copies)(1q21)^f, g, i, k^	MCL1/CKS1B/ANP32E/BCL9	35–40%				
del(6q,8p,13q14^j, k^,13^h,j^, 11q, 12p,14q,16q)^f^	RB1/DIS3/BIRC2 BIRC3/TRAF3/WWOX CYLD/EBPL/CD27/mir	7–44%^e^				

*amp* Amplification, *ANP32E* Acidic nuclear phosphoprotein 32 family member E, *BCL9* B-cell lymphoma 9, *BIRC* Baculoviral IAP repeat-containing, *CA* Cytogenetic abnormality, *CCND* Cyclin D, *CDKN2C* Cyclin-dependent kinase inhibitor 2C, *CKS1B* Cyclin-dependent kinase regulatory subunit 1B, *Cx* Chromosome, *CYLD* Cylindromatosis lysine 63 deubiquitinase, *del* Deletion, *DIS3* Defective in sister chromatid joining 3, *EBPL* Emopamil-binding protein-like, *FAF1* FAS-associated factor 1, *FAM46C* Family with sequence similarity 46 member C, *FGFR3* Fibroblast growth factor receptor 3, *MAF* v-maf musculoaponeurotic fibrosarcoma oncogene homolog, *MCL1* Myeloid cell leukemia sequence 1, *MMSET* Multiple myeloma SET domain, *NA* Not available, *RB1* Retinoblastoma 1, *t(x;y)* Chromosomal translocation (x,y), *TP53* Tumor protein P53, *TRAF3* Tumor necrosis factor receptor associated factor 3, *WWOX* WW domain-containing oxidoreductase

^a^TP53 locus; ^b^3,5,7,9,11,15,19; ^c^IgH translocations; ^d^primary genetic events in multiple myeloma consisting of the IgH translocation group and the hyperdiploidy group; ^e^6q (33%), 8p (25%), 13 (44%), 11q (7%), 12p (15%), 14q (38%), 16q (35%); ^f^secondary or progression genetic events in multiple myeloma consisting of the deletion and gain group; ^g^high-risk cytogenetic abnormalities according to the R2-ISS (Second Revision of the International Staging System); ^h^intermediate-risk cytogenetic abnormalities according to the (updated) mSMART criteria (Mayo Clinic Risk Stratification for multiple myeloma, 20%); ^i^high-risk cytogenetic abnormalities according to the (updated) mSMART criteria (Mayo Clinic Risk Stratification for multiple myeloma, 20%); ^j^in chromosome 13 changes, a monosomy 13 accounts for 85–90% of alterations and del(13q14) for 10–15%. All chromosome 13 alterations are strongly correlated with other high-risk genetic features such as t(4;14)(p16;q32), del(17p13), or high serum β2-microglobulin; ^k^high-risk cytogenetic abnormalities according to the IMWG (International Myeloma Working Group) 2016 Consensus Statement on treatment of multiple myeloma with high-risk cytogenetics

### Imaging

**Fig. 2:**
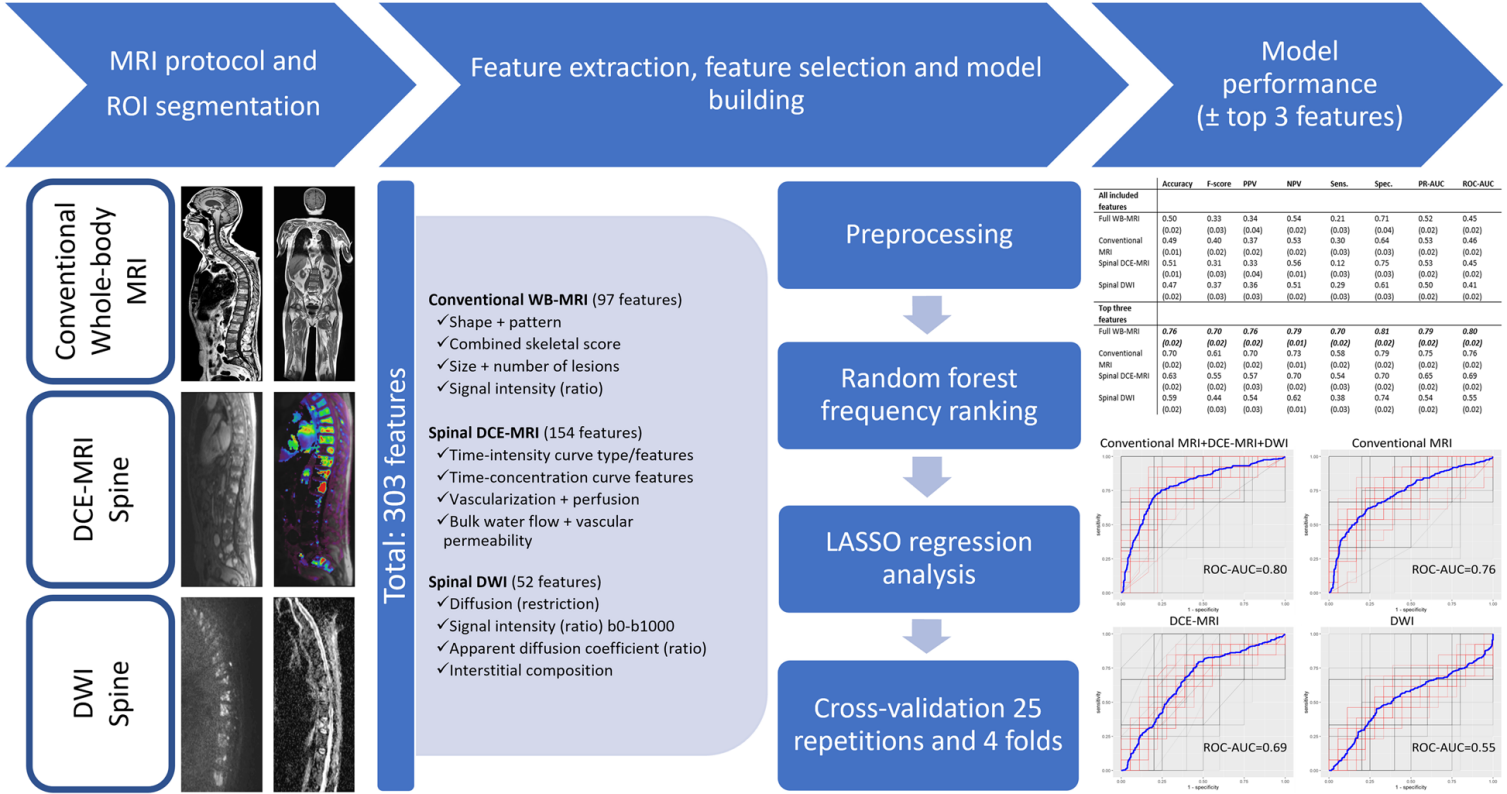
General overview of the MRI protocol and of the methods used for region-of-interest segmentation on the conventional anatomical whole-body MRI, spinal dynamic contrast-enhanced MRI, and spinal diffusion-weighted MRI sequences, for feature extraction, for feature selection, for statistical model building and for testing the models’ performances. In general, models are tested using receiver operating characteristic curve analysis including all MRI features and separate models are retested on the dataset using only the top three most predictive MRI features (in the final model with the three most prevalent features, generalizability can be reduced due to lack of external testing). *AUC* area-under-the-curve, *b0-b1000* diffusion sensitizing gradients, *DCE* dynamic contrast-enhanced, *DWI* diffusion-weighted imaging, *LASSO* least absolute shrinkage and selection operator, *MRI* magnetic resonance imaging, *NPV* negative predictive value, *PPV* positive predictive value, *PR-AUC* precision-recall area-under-the-curve, *ROC* receiver operating characteristic, *ROI* region-of-interest, *sens.* sensitivity, *spec.* specificity, *WB* whole-body

From imaging, an overview of the methods is described in Fig. 2.

**Fig. 3:**
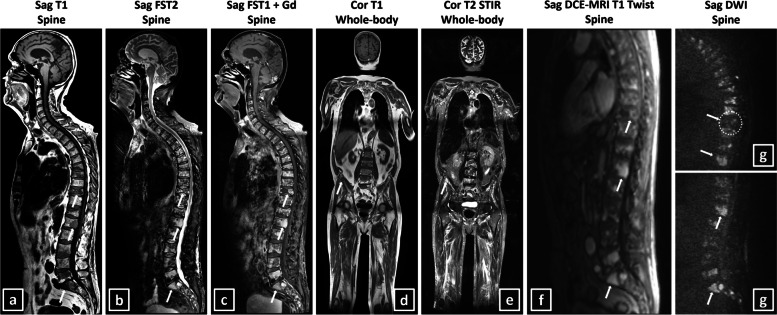
Overview of the 1.5-Tesla MRI scanning protocol using whole-body conventional anatomical MRI sequences (**a**–**e**), spinal dynamic contrast-enhanced MRI (**f**), and spinal diffusion-weighted imaging (**g**). A 77-year-old male patient with double hit high-risk IgGκ multiple myeloma with Salmon-Durie Plus and Revised International Staging System (second revision, R2-ISS) stadium II is presented, which was unresponsive to therapy and passed away 1.8 years after diagnosis. Regarding SLIM-CRAB criteria, a monoclonal bone marrow plasmacytosis of 50%, a light-chain involved/uninvolved ratio of 42, a total number of 19 focal MRI lesions > 5 mm (largest: 22 mm), a normocalcaemia, a mildly reduced renal function (glomerular filtration rate 60 mL/min/1.72 m^2^, CKD stage G2) and a macrocytic anemia were observed. Suspected focal lesions of more than 5 mm in the 10th thoracic, 1st lumbar, and 1st sacral vertebra and the right iliac bone (white arrows) and diffuse abnormal signal intensities on all sequences are observed. A combined skeletal score of 11/13 with a combined focal and diffuse bone marrow invasion pattern can be observed. The b1000 diffusion-weighted images show severe diffusion restriction in all vertebrae and in the focal lesions of the 1st lumbar and 1st sacral vertebra (white arrows in **g**). The suspected focal lesion in the 10th thoracic vertebra does not show diffusion restriction (white dotted circle) or contrast enhancement, depicting its benign character due to a recent compression fracture. The spinal dynamic contrast-enhanced MRI sequence, 50 s after gadolinium contrast administration (Gadovist 7.5 mL, gadobutrol 1.0 mmol/mL, 0.1 mmol/kg, Bayer), shows intense and fast contrast uptake in the entire spine and especially in the focal lesions (white arrows in **f**). *Cor* coronal, *DCE-MRI* dynamic contrast-enhanced magnetic resonance imaging, *DWI* diffusion-weighted imaging, *FS* fat-saturated, *Gd* gadolinium, *sag* sagittal, *STIR* short tau inversion recovery, *T1* T1-weighted, *T2* T2-weighted

All patients were scanned with multiple surface coils in a supine position with head first and hands positioned at the sides of the body on a 1.5-Tesla MRI machine (Magnetom AvantoFit-Siemens) with a 90-min (un)enhanced conventional anatomical whole-body MRI (sagittal sequences head-coccygeal spine, coronal sequences head-proximal tibia), spinal DCE-MRI, and spinal DWI protocol (thoracic-coccygeal spine) (overview and technical information: Table 3, Fig. 3) [40].

**Table 3:** MRI scanning protocol and technical parameters

	**Conventional anatomical MRI**					**Functional MRI**	
	**CorT1w** **TSE**	**CorT2w** **STIR**	**SagT1w** **TSE**	**SagFST2w** **TSE**	**SagFST1w** **TSE + Gd**^**a**^	**Sag DCE-MRI**^**a, b**^	**Sag DWI**^**c**^
**Region**	WB (head-proximal tibia)	WB (head-proximal tibia)	Head-coccygeal spine	Head-coccygeal spine	Head-coccygeal spine	Thoracic-coccygeal spine	Thoracic-coccygeal spine
**TR (ms)**	661	8640	576	7270	771	4.25	7700
**TE (ms)**	8.8	108	10	68	10	1.73	86
**TI (ms)**	N/A	140	N/A	N/A	N/A	N/A	180
**ST (mm)**	7	7	3.3	3.3	3.3	4	3
**Spacing (mm)**	7	7	3.3	3.3	3.3	4	3.3
**Type**	2D	2D	2D	2D	2D	3D	2D
**Averages**	3	1	2	1	2	1	3
**ETL**	3	21	3	13	3	0	89
**Pixel BW**	215	130	165	130	165	280	1530
**AM**	0/384/384/0	0/384/384/0	384/0/0/288	384/0/0/384	384/0/0/288	0/192/138/0	0/192/192/0
**Flip angle**	150	150	150	150	150	12	90
**PS**	1.30/1.30	1.30/1.30	0.91/0.91	0.91/0.91	0.91/0.91	2.34/2.34	1.67/1.67
**FOV**	501*1289	501*1285	777*351	777*350	777*350	450*450	319*319
***b*-values**	N/A	N/A	N/A	N/A	N/A	N/A	0–200–400–600–1000

*2D* Two-dimensional, *3D* Three-dimensional, *AM* Acquisition matrix, *b-values* Diffusion sensitizing gradients of the diffusion-weighted imaging sequence, *BW* Bandwidth, *cor* Coronal, *DCE* Dynamic contrast-enhanced, *DWI* Diffusion-weighted imaging, *EPI* Echo planar imaging, *ETL* Echo train length, *FOV* Field of view, *FS* Fat-saturated, *Gd* Gadolinium, *mm* Millimeter, *MRI* Magnetic resonance imaging, *ms* Millisecond, *N/A* Not available, *PS* Pixel spacing, *sag* Sagittal, *ST* Slice thickness, *STIR* Short tau inversion recovery, *T1w* T1-weighted, *T2w* T2-weighted, *TE* Echo time, *TI* Inversion time, *TR* Repetition time, *TSE* Turbo spin-echo, *WB* Whole-body

^a^The injection rate of contrast agent was 3–5 mL/s. Contrast agent used was Gadovist (gadobutrol 1.0 mmol/mL, 0.1 mmol/kg, Bayer), Dotarem (gadoteric acid 0.5 mmol/mL, 0.1 mmol/kg, Guerbet), and Magnevist (gadopentetate dimeglumine 0.5 mmol/mL, 0.1 mmol/kg, Bayer) in 23 (= 74%), one (= 3%), and seven (= 23%) patients, respectively. In general, a 3D Twist-Vibe sequence was used. Before the dynamic sequence, a sagittal T1 vibe sequence with variable flip angle was performed (2° and 15°). After gadolinium injection, 74 times eight parallel fat-suppressed T1-weighted multi-slice sagittal 3D images were acquired covering the thoracic to coccygeal spine with an interval of 1600 ms for 2 min

^b^In Siemens SyngoViaVB60 (MROncology and MRTissue4D reading and postprocessing modules), the dynamic images were analyzed according to the software’s protocol for DCE-MRI. Regarding preprocessing, homogenization, and normalization of images, both motion correction and elastic alignment of the pre-contrast to the dynamic series were applied. Afterward, in the processing steps, two pharmacokinetic models were available, one for qualitative and semiquantitative assessment and one for quantitative assessment (Tofts model). In both steps, the model depends on the contrast agent used because they have specific characteristics like relaxivity [L/mmol/s], molarity [mmol/mL], dose [mmol/kg], and injected volume. Also, both models depend on the arrival time of contrast agent [s], which is set manually based on the interpretation of the time-intensity curve within the region of interest. In the qualitative model, the protocol uses a constant T1 value, which was set to 2000 ms with a threshold of 20 ms. In the Tofts model, an arterial input function (slow, intermediate, or fast) is used based on the performance of the function to model the raw data points of the time-concentration curve. The difference or error rate between the function and the raw data points is called the chi square metric. The arterial input function with the smallest chi square is used for further analysis. A pixelwise T1 protocol is used with a threshold of 20 ms

^c^Technique used: echo planar imaging using different diffusion-sensitizing gradients or *b*-values (0–200–400–600–1000) with calculation of the corresponding apparent diffusion coefficient maps using all *b*-values images

### Image reading as index test

The images were analyzed by two radiologists (T.V.D.B., in-training/KLV) in consensus (to increase the quality of readings and measurements) with 4/33 years of experience in musculoskeletal MRI after initial training, reading, and segmentation sessions. The readers were blinded for disease characteristics and genetic tests [54]. Training consisted of both qualitative scoring and quantitative measuring sessions for both readers according to the latest state-of-the-art scientific and practical background information. All image readings, interpretations, qualitative analyses, (semi)quantitative analyses, and manual segmentations were performed by both readers in consensus (four-eye principle) in Siemens SyngoViaVB60 (MROncology and MRTissue4D reading and postprocessing modules).

**Fig. 4:**
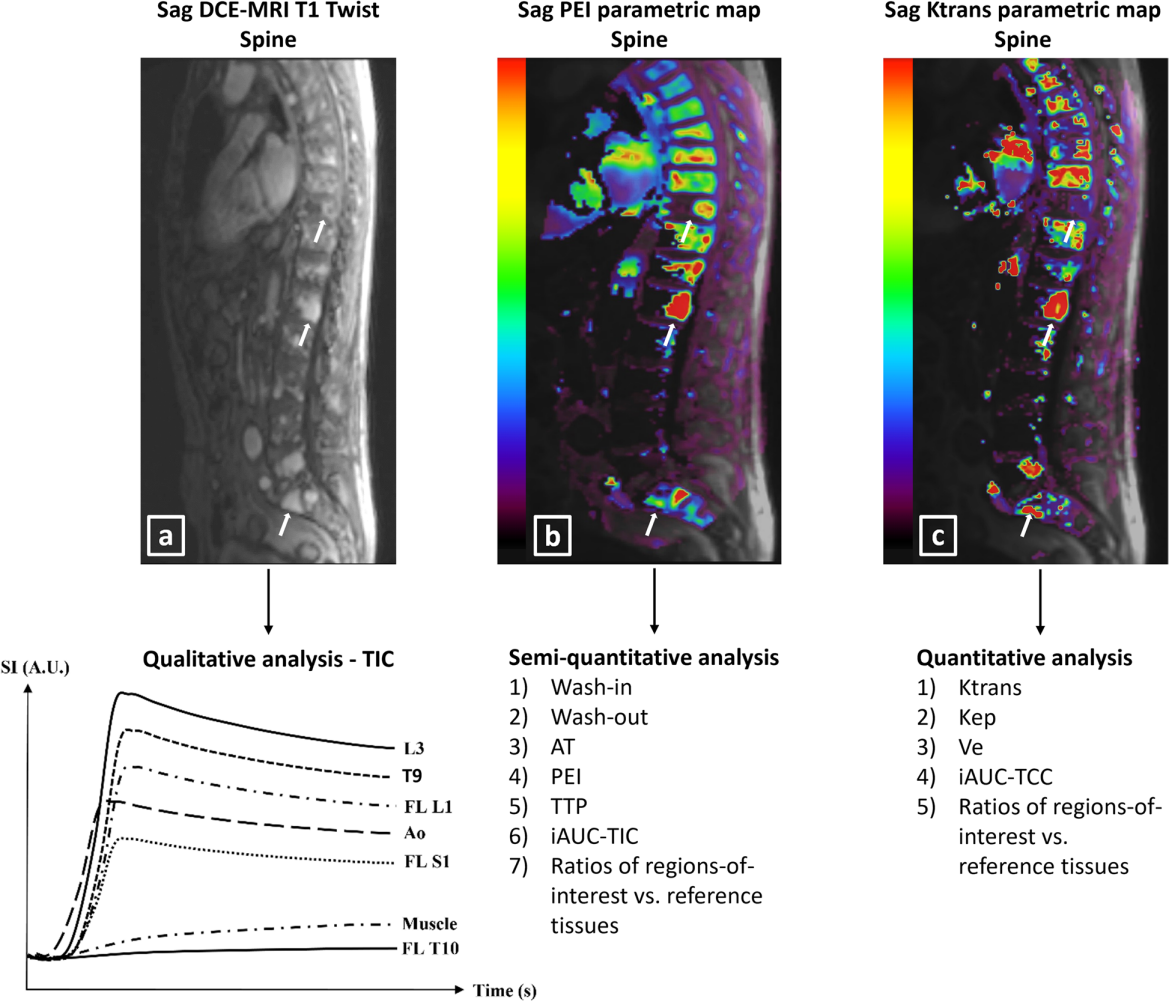
Assessment of spinal dynamic contrast-enhanced MRI to obtain qualitative time-intensity curves (**a**), semi-quantitative (**b**), and quantitative (**c**) parametric maps and features of regions-of-interest in the spine and of reference tissues in the same patient as in Fig. 3. Cortical endplates, basivertebral veins, normal anatomical variants, and benign lesions like Schmorl’s nodules and Modic changes were avoided during segmentation. Regions-of-interest and reference tissue segmentations were matched with the anatomical sequences for optimal detailed segmentation. **a** Suspected focal lesions ≥ 5 mm in the 10th thoracic, 1st lumbar, and 1st sacral vertebra (arrows on the sagittal spinal dynamic contrast-enhanced MRI T1 Twist sequence, 50 s after gadolinium contrast administration) (Gadovist 7.5 mL, gadobutrol 1.0 mmol/mL, 0.1 mmol/kg, Bayer) and diffuse abnormal signal intensities can be observed in the spinal bone marrow. On the derived time-intensity curve, the thoracic and lumbar vertebral bone marrow (L3-third lumbar vertebra; T9-ninth thoracic vertebra) show active type IV curves with a steep first pass corresponding to high perfusion, high tissue vascularization, and low capillary resistance. The steep wash-in of a type IV curve and strong wash-out depict the effect of a highly vascularized region in combination with a small interstitial space. The suspected focal lesions in the 1st lumbar (FL L1) and 1st sacral (FL S1) vertebrae also show active type IV curves. The suspected focal lesion in the 10th thoracic (FL T10) vertebra shows an inactive type I curve without enhancement which is comparable to the reference paravertebral muscle vascularization, indicative of its benign character due to a recent compression fracture. Remark that the diffuse bone marrow infiltration also shows a type IV curve, indicative that active myeloma disease invades the entire spine diffusely. **b** Sagittal spinal positive enhancement integral parametric map generated in SyngoVia VB60 (Siemens) postprocessing software to assess the semi-quantitative features describing the time-intensity curve. Extracted features are wash-in, wash-out, arrival time, positive enhancement integral, time-to-peak, and initial area-under-the-time-intensity-curve (60 s). E.g. the positive enhancement integral is low (0.033) in the paravertebral muscles as reference tissue. The bone marrow of the ninth thoracic vertebral body and the focal lesion in the first lumbar vertebra have a positive enhancement integral of 0.244 and 0.441, respectively, which is 7–13 times higher than that of the reference muscle. **c** Sagittal spinal Ktrans (volume transfer constant) parametric map generated in SyngoVia VB60 (Siemens) postprocessing software to assess the quantitative features resulting from the Tofts model describing the time-concentration curve. Extracted features are Ktrans (volume transfer constant), Kep (rate constant), Ve (volume of the extracellular extravascular space), and initial area-under-the-time-concentration-curve (60 s). E.g. the Ktrans is low (0.094) in the paravertebral muscles as reference tissue. The bone marrow of the ninth thoracic vertebral body and the focal lesion in the first lumbar vertebra have a Ktrans of 1.094 and 1.494, respectively, which is 12–16 times higher than that of the reference muscle. *Ao* aorta, *AT* arrival time, *A.U.* arbitrary unit, *DCE-MRI* dynamic contrast-enhanced magnetic resonance imaging, *FL* focal lesion,* iAUC* initial area-under-the-curve, *Kep* rate constant, *Ktrans* volume transfer constant, *L1/L3* first/third lumbar vertebra, *PEI* positive enhancement integral, *s* second, *S1* first sacral vertebra, *sag* sagittal, *SI* signal intensity, *T1* T1-weighted, *T9/T10* ninth/tenth thoracic vertebra, *TCC* time-concentration curve, *TIC* time-intensity curve, *TTP* time-to-peak, *Ve* volume of the extracellular extravascular space, vs. versus

Regarding spinal DCE-MRI, segmentations of the centers of the largest focal lesion and of the third lumbar (L3) and of the tenth thoracic vertebral bodies (T10) which were free of focal lesions (= normal appearance or diffusely involved) were performed as regions-of-interest for perfusion analysis. If the L3 and/or T10 vertebral bodies were focally involved with MM, an adjacent vertebral body free of focal lesions was used for perfusion analysis. Moreover, segmentations of the center of the aorta (without flow artifacts) and of a fat-free region of a paravertebral muscle as reference tissues were performed. A time-intensity curve (TIC) was plotted for all segmented regions. A qualitative classification of five curve types to assess vascularization in all regions-of-interest was performed [40]. The vascularization of the thoracic and lumbar spine was scored separately and categorized as steep/highly perfused (types III/IV/V) or slow/lowly perfused (types I/II). Semi-quantitative TIC analysis of all regions-of-interest extracted absolute features [wash-in(WI)/wash-out(WO)/arrival time(AT)/positive enhancement integral(PEI)/time-to-peak(TTP)/initial-area-under-curve(iAUC-TIC)]. A quantitative analysis in all regions-of-interest was performed using the modified Tofts model [39, 55–57]. Time-concentration curves (TCC) of the regions-of-interest and reference tissues were generated, defining absolute features describing the concentration distribution of gadolinium over the vascular and interstitial compartments: Ktrans/Kep/Ve/iAUC-TCC [39, 57–59]. For all features, ratios of values of regions-of-interest relative to reference tissues were calculated [wash-in ratio (WIR)/wash-out ratio (WOR)/arrival time ratio (ATR)/positive enhancement integral ratio (PEIR)/time-to-peak ratio (TTPR)/initial-area-under-the-time-intensity-curve ratio (iAUC-TICR)/KtransR/KepR/VeR/initial-area-under-the-time-concentration-curve ratio (iAUC-TCCR)] (Fig. 4) [40].

Regarding spinal DWI, the mean signal intensity (SI) was measured on b0 and b1000 images in segmentations in the centers of the largest focal lesion and of the T10 and L3 vertebral bodies. A homogeneous area in the spinal medulla and an area without flow artifacts in the cerebrospinal fluid (CSF) at the L4 level were used as reference tissues. b0 and b1000 ratios of the mean SI of the regions-of-interest relative to reference tissues were calculated (b0R and b1000R). The bslope was calculated ($bslope=\frac{SIb1000-SIb0}{1000}$) for the regions-of-interest and reference tissues. The bslope ratio(bslopeR) was calculated by dividing the bslope of the regions-of-interest by that of the reference tissues. Apparent diffusion coefficients (ADC) and ADC-maps using all five *b*-value images (0–200–400–600–1000) were calculated. ADC ratios (ADCR) of the ADC of regions-of-interest relative to reference tissues were calculated [39, 60, 61]. Moreover, *b*-value images of 1000 s/mm^2^ were classified as “normal” or “abnormal” (= “increased diffusion restriction”) and a qualitative score was applied (0 = normal/1 = mild diffusion restriction/2 = moderate diffusion restriction/3 = severe diffusion restriction) [37] (Fig. 5).

**Fig. 5:**
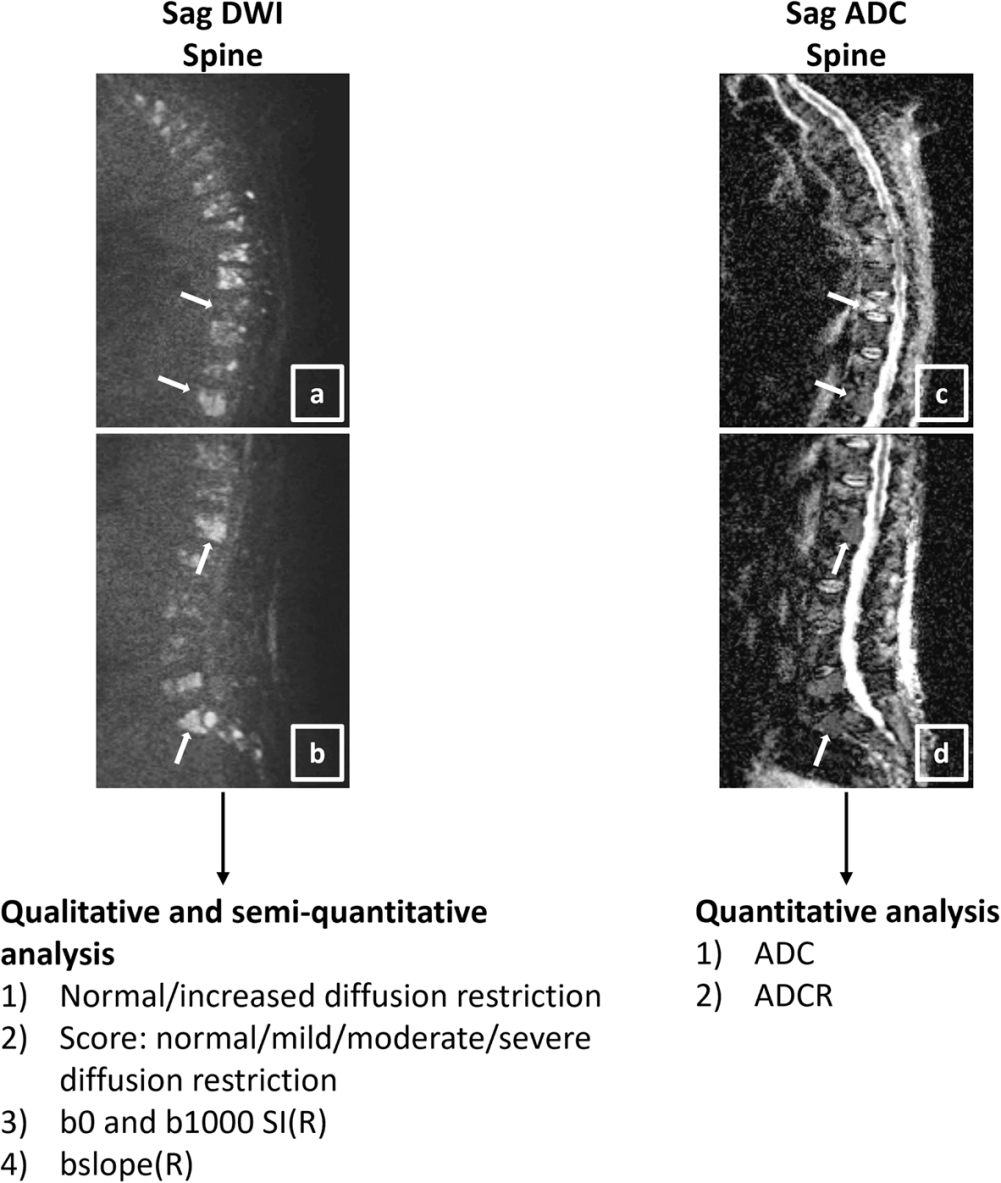
Assessment of spinal diffusion-weighted imaging (**a**, **b** b1000 thoracic (**a**) and lumbar (**b**) spine images) to obtain a qualitative and (semi-)quantitative interpretation of the diffusion restriction of regions-of-interest in the spine and of reference tissues in the same patient as in Fig. 3. For the apparent diffusion coefficients and corresponding parametric maps (thoracic (**c**) and lumbar (**d**) spine), all *b*-values (0, 200, 400, 600, 1000) were used for analysis. Regions-of-interest and reference tissue segmentations were matched with the anatomical sequences for optimal detailed segmentation. E.g. the apparent diffusion coefficient of the ninth thoracic vertebra (diffusely invaded bone marrow), of the tenth thoracic vertebra (benign compression fracture), of the focal lesion in the first lumbar vertebra, and of the focal lesion in the first sacral vertebra (white arrows) equal 712, 1330, 801, and 658 × 10^-6^ mm^2^/s, indicating diffusion restriction in all regions-of-interest except for the benign compression fracture in the tenth thoracic vertebra. *ADC(R)* apparent diffusion coefficient (ratio), *bslope(R)* bslope (ratio), *b-value* diffusion-sensitizing gradient, *DWI* diffusion-weighted imaging, *sag* sagittal, *SI(R)* signal intensity (ratio)

Evaluation of BM involvement on conventional anatomical whole-body MRI was achieved using the “combined skeletal score” (= number of affected skeletal regions = x/13) [36, 40]. The pattern of BM invasion was analyzed. A dichotomous separation was made between focal disease only and other types of BM invasion. Next, BM invasion was scored as focal/salt-and-pepper/diffuse/diffuse and focal or salt-and-pepper [39, 62–66]. Focal lesions > 5 mm were counted and the diameter/volume of the largest focal lesion was measured. Mean SI was measured on all sagittal sequences in the centers of the T10/L3 vertebral body and spinal process and in the largest focal lesion. An area without flow artifacts of lumbar CSF, a fat-free region of paravertebral muscle, and the center of a non-degenerative intervertebral disc were used as reference tissues. On coronal sequences, the mean SI in the center of the left and right coracoid process and suprasternal notch were measured. SI ratios (SIR) of the SI of the regions-of-interest relative to that of the reference tissues (same anatomical level) were calculated to eliminate the distance to the MRI coils effect.

### Feature selection and model building

To discover (combinations of) features that are discriminative for genetic risk, both univariate and model-based methods were performed (S.W., statistician with 4 years’ experience). For univariate analyses, Wilcoxon rank sum tests were performed [67].

For the model-based analyses, a pipeline was set up for feature and model selection. After preprocessing, the feature selection was performed based on the frequency and unique values ratios. Next, a random forest was trained (500 trees). A ranking of the features was obtained after which the most predictive features were selected. To balance cases in both genetic risk classes, adaptive synthetic sampling for imbalanced learning (ADASYN) was applied [68]. Different linear (logistic least absolute shrinkage and selection operator-LASSO) and nonlinear (random forests/radial basis function kernel support vector machines/neural networks) classification methods were explored without extensive hyperparameter tuning, showing similar performances. Logistic LASSO as feature selection method was used to delete redundant or strongly correlated features.

The pipeline contained two tunable hyperparameters, which were optimized simultaneously (= Bayesian): percentage of features to select in the random forest feature selection step and the LASSO penalty parameter.

A 25 times repeated stratified *k*-fold cross-validation was performed to estimate the statistical model’s performance (accuracy/F-score/negative predictive value (NPV)/precision-recall area-under-the-curve (PR-AUC)/positive predictive value (PPV)/sensitivity/receiver-operating-characteristic AUC(ROC-AUC)/specificity). Bootstrapping (B = 25) nested within each fold to cross-validate the hyperparameter tuning was performed. A *k* = 4 was chosen (balance in training and test set: 75–25% split).

The performance of four different models was tested including (1) all features of the entire multiparametric MRI examination, (2) all conventional anatomical MRI features only, (3) all DCE-MRI features only, and (4) all DWI features only. As a final step, four final models were tested with the three most predictive features [69].

Analyses were performed with R4.2.2 (Microsoft Corporation). *p* < 0.05 was considered statistically significant, and *p* < 0.001 was considered strongly significant (Supplementary Materials/Supplemental Fig. 1: detailed statistics).

## Results

### Study group, clinical parameters, and genetic analysis

Thirty-one patients (mean age = 66.4 ± 7.4 years, 15 men) were enrolled after patient selection and exclusion (Fig. 1). Thirteen patients had intermediate-/high-risk (mean age = 68.0 ± 6.4 years, six men, 2-year OS = 92%, 3-year OS = 92%) and 18 had standard-risk cytogenetics (mean age = 65.3 ± 8.1 years, nine men, 2-year OS = 100%, 3-year OS = 94%). Regarding risk stratification, 18/13 patients were classified as Salmon-Durie plus (SDP) stadium I/II and 16/15 patients as R2-ISS stadium I/II (Table 1) [26, 40, 41].

### Imaging, image reading, and MRI features

In total, 303 MRI features were extracted from all MRI sequences. From the conventional anatomical/DCE-/DWI-MRI studies, 97/154/52 features were extracted, respectively.

The combined skeletal score was 9/13 in both CA risk groups. A purely focal BM invasion pattern was only observed in the intermediate-/high-risk group. More and larger focal lesions were present in the intermediate-/high-risk group. No differences between intermediate-/high-risk and standard-risk groups were observed concerning DCE-MRI and DWI.

In the thoracic spine, 6/25 patients had a slow/steep TIC slope. In the lumbar spine, 11/20 patients had a slow/steep TIC slope. In the thoracic spine, 7/24 patients had a normal/increased diffusion restriction. In the lumbar spine, 9/22 patients had a normal/increased diffusion restriction (Table 4).

**Table 4:** Descriptive general MRI features of the conventional anatomical whole-body MRI, spinal dynamic contrast-enhanced MRI, and spinal diffusion-weighted imaging of the entire patient population, the intermediate-/high-, and the standard-risk cytogenetic group

	**All patients (*n* = 31, 100%)**	**Intermediate-/high-risk (*n* = 13, 42%)**	**Standard-risk (*n* = 18, 58%)**
**Conventional WB-MRI**			
Contrast	23 Gadovist (74%)	12 Gavovist (92%)	11 Gadovist (61%)
	1 Dotarem (3%)		1 Dotarem (6%)
	7 Magnevist (23%)	1 Magnevist (8%)	6 Magnevist (33%)
Skeletal score	9.0 ± 2.9	9.0 ± 3.0	9.0 ± 2.9
Pattern general	2 focal only (6%)	2 focal only (15%)	0 focal only (0%)
	29 generalized (94%)	11 generalized (85%)	18 generalized (100%)
Pattern specific	2 focal (6%)	2 focal (15%)	0 focal (0%)
	2 salt-and-pepper (6%)	0 salt-and-pepper (0%)	2 salt-and-pepper (11%)
	8 diffuse (26%)	2 diffuse (15%)	6 diffuse (33%)
	19 focal + diffuse/salt-and-pepper (62%)	9 focal + diffuse/salt-and-pepper (70%)	10 focal + diffuse/salt-and-pepper (56%)
FL	21 present (68%)	11 present (85%)	10 present (56%)
	10 absent (32%)	2 absent (15%)	8 absent (44%)
FL number	18: 0–4 (58%)	5: 0–4 (38%)	13: 0–4 (72%)
	13: 5–20 (42%)	8: 5–20 (62%)	5: 5–20 (28%)
	0: > 20 (0%)	0: > 20 (0%)	0: > 20 (0%)
Largest FL diameter (mm)	21.9 ± 21.8	23.1 ± 28.9	20.8 ± 13.0
Largest FL volume (mm^3^)	33.6 ± 116.2	56.7 ± 164.4	10.4 ± 17.9
**Spinal DCE-MRI**			
TIC type thoracic general	6 slow^a^ (19%)	3 slow^a^ (23%)	3 slow^a^ (17%)
	25 steep^b^ (81%)	10 steep^b^ (77%)	15 steep^b^ (83%)
TIC type thoracic specific	2: I (7%)-4: II (13%)-6: III (19%)-19: IV (61%)-0: V (0%)	1: I (8%)-2: II (15%)-4: III (31%)-6: IV (46%)-0: V (0%)	1: I (6%)-2: II (11%)-2: III (11%)-13: IV (72%)-0: type V (0%)
TIC type lumbar general	11 slow^a^ (35%)	5 slow^a^ (38%)	6 slow^a^ (33%)
	20 steep^b^ (65%)	8 steep^b^ (62%)	12 steep^b^ (67%)
TIC type lumbar specific	7: I (23%)-4: II (13%)-6: III (19%)-13: IV (42%)-1: V (3%)	4: I (31%)-1: II (7%)-4: III (31%)-4: IV (31%)-0: V (0%)	3: I (17%)-3: II (17%)-2: III (11%)-9: IV (50%)-1: V (5%)
**Spinal DWI**			
Restriction thoracic general	7 normal (23%)	3 normal (23%)	4 normal (22%)
	24 increased (77%)	10 increased (77%)	14 increased (78%)
Increased restriction thoracic specific	7 normal (23%)	3 normal (23%)	4 normal (22%)
	8 mild (25%)	4 mild (31%)	4 mild (22%)
	9 moderate (29%)	3 moderate (23%)	6 moderate (34%)
	7 severe (23%)	3 severe (23%)	4 severe (22%)
Restriction lumbar general	9 normal (29%)	4 normal (31%)	5 normal (28%)
	22 increased (71%)	9 increased (69%)	13 increased (72%)
Increased restriction lumbar specific	9 normal (29%)	4 normal (31%)	5 normal (28%)
	13 mild (42%)	5 mild (38%)	8 mild (44%)
	4 moderate (13%)	1 moderate (8%)	3 moderate (17%)
	5 severe (16%)	3 severe (23%)	2 severe (11%)

*DCE-MRI* Dynamic contrast-enhanced MRI, *DWI* Diffusion-weighted imaging, *FL* Focal lesion, *mm* Millimeter, *mm*^*3*^ Cubic millimeter, *n* Number, *TIC* Time-intensity curve, *WB* Whole-body

^a^Slow = time-intensity curve type I and II

^b^Steep = time-intensity curve type III, IV, V

### Feature selection and model building

In univariate analysis, the MRI-based genetic risk prediction identified eight significant features (unadjusted *p* < 0.05) but none of them showed significance after statistical correction. So, no single MRI-parameter alone could predict cytogenetic risk.

The statistical outcome of the model-based analyses of the four general models (all included features) and four final models (three most predictive features) is summarized in Table 5. For the multiparametric MRI protocol with all sequences included, the three most predictive features were SIR T2w between the L3 spinous process and the CSF, SIR T1w between the largest spinal focal lesion and the CSF and b1000R between the L3 vertebral body and the CSF. For the conventional anatomical MRI sequence only, the three most predictive features were SIR T2w between the L3 spinous process and the CSF, SIR T2w between the L3 spinous process and the intervertebral disc, and SIR T1w between the largest spinal focal lesion and the CSF. For the DCE-MRI sequence only, the three most predictive features were PEIR between T10 and L3, WOR between T10 and muscle, and iAUC-TICR between T10 and muscle. For the DWI sequence only, the three most predictive features were b0R between T10 and L3, b1000R between T10 and L3 and b1000 of L3.

**Table 5:** Mean statistical performance metrics for all repeats and folds with standard error between brackets for the four general models with all included features and four final models with three included features which were chosen most frequently for every model (= most predictive features for cytogenetic risk classification for every MRI sequence separately)

	**Accuracy**	***F*-score**	**PPV**	**NPV**	**Sens**	**Spec**	**PR-AUC**	**ROC-AUC**
**All included features**								
Full WB-MRI	0.50 (0.02)	0.33 (0.03)	0.34 (0.04)	0.54 (0.02)	0.21 (0.03)	0.71 (0.04)	0.52 (0.02)	0.45 (0.02)
Conventional anatomical MRI	0.49 (0.01)	0.40 (0.02)	0.37 (0.02)	0.53 (0.02)	0.30 (0.03)	0.64 (0.03)	0.53 (0.02)	0.46 (0.02)
Spinal DCE-MRI	0.51 (0.01)	0.31 (0.03)	0.33 (0.04)	0.56 (0.01)	0.12 (0.03)	0.75 (0.03)	0.53 (0.02)	0.45 (0.02)
Spinal DWI	0.47 (0.02)	0.37 (0.03)	0.36 (0.03)	0.51 (0.02)	0.29 (0.03)	0.61 (0.03)	0.50 (0.02)	0.41 (0.02)
**Top three features**								
Full WB-MRI	***0.76 (0.02)***	***0.70 (0.02)***	***0.76 (0.02)***	***0.79 (0.01)***	***0.70 (0.02)***	***0.81 (0.02)***	***0.79 (0.02)***	***0.80 (0.02)***
Conventional anatomical MRI	0.70 (0.02)	0.61 (0.02)	0.70 (0.02)	0.73 (0.01)	0.58 (0.02)	0.79 (0.02)	0.75 (0.02)	**0.76 (0.02)**
Spinal DCE-MRI	0.63 (0.02)	0.55 (0.02)	0.57 (0.03)	0.70 (0.02)	0.54 (0.03)	0.70 (0.02)	0.65 (0.02)	**0.69 (0.02)**
Spinal DWI	0.59 (0.02)	0.44 (0.03)	0.54 (0.03)	0.62 (0.01)	0.38 (0.03)	0.74 (0.02)	0.54 (0.02)	**0.55 (0.02)**

*AUC* Area-under-the-curve, *DCE* Dynamic contrast-enhanced, *DWI* Diffusion-weighted imaging, *LASSO* Least absolute shrinkage and selection operator, *MRI* Magnetic resonance imaging, *NPV* Negative predictive value, *PPV* Positive predictive value, *PR* Precision-recall, *ROC* Receiver operating characteristic, *sens.* Sensitivity, *spec.* Specificity, *WB* Whole-body

**Fig. 6:**
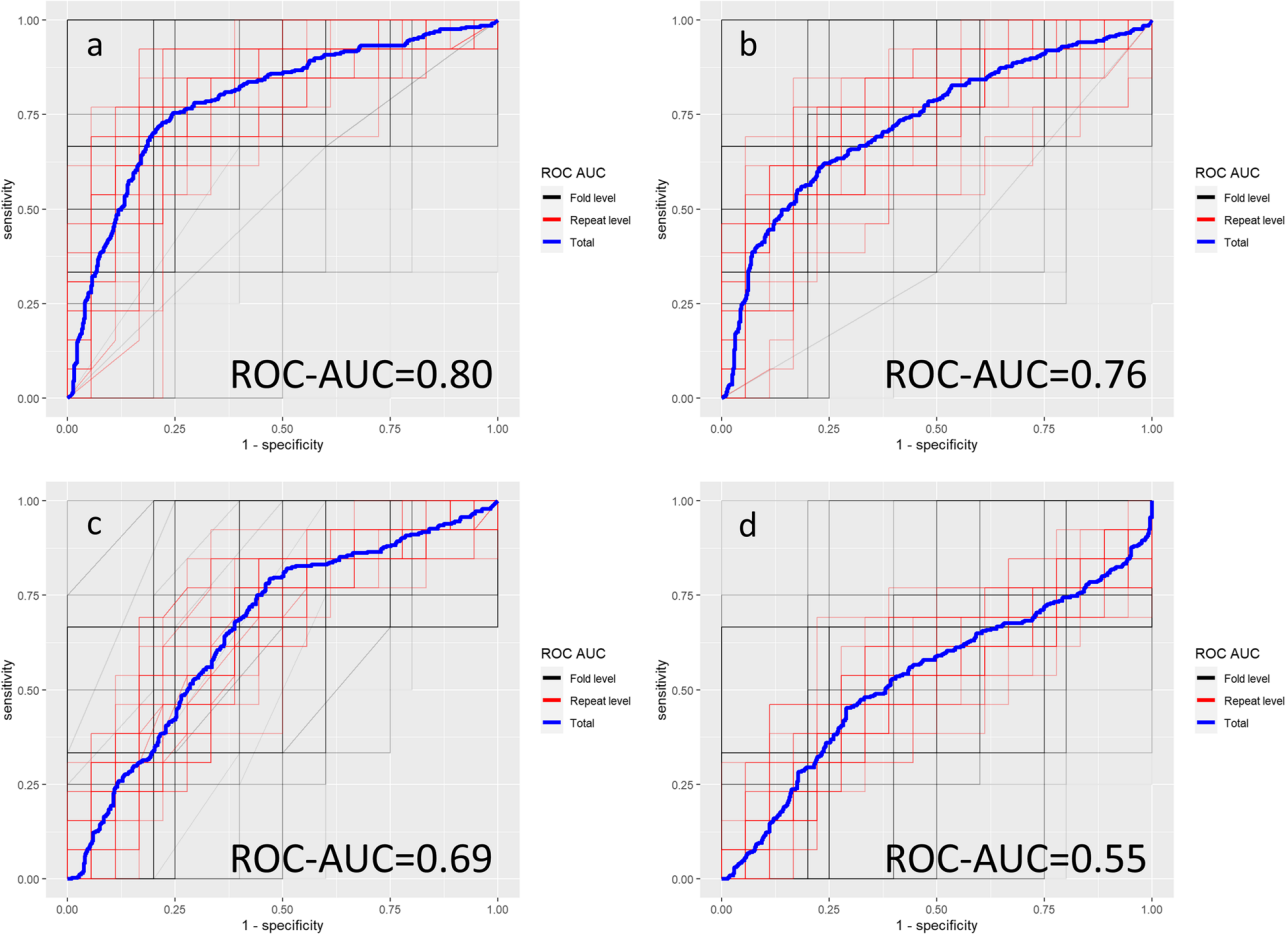
Receiver operating characteristic (ROC) curves for the four final models based upon the three most frequently LASSO-selected features (= most predictive features for cytogenetic risk classification). **a** In the entire multiparametric MRI protocol including all sequences (conventional anatomical whole-body MRI + spinal dynamic contrast-enhanced MRI + spinal diffusion-weighted imaging). **b** In the conventional anatomical whole-body MRI sequence only. **c** In the spinal dynamic contrast-enhanced MRI sequence only. **d** In the spinal diffusion-weighted MRI sequence only. Overall statistical performance is expressed by the ROC-AUC (receiver operating characteristic area-under-the-curve)

In the final model with the three most predictive features, a ROC-AUC 0.80, PR-AUC 0.79, sensitivity 0.70, specificity 0.81, PPV 0.76, NPV 0.79, accuracy 0.76, and F-score 0.70 were obtained for the entire multiparametric MRI examination including all sequences. All statistical metrics reached highest performance when all three MRI techniques were combined, where the statistical performance of the conventional anatomical MRI separately always exceeded that of DCE-MRI or DWI separately and the performance of DCE-MRI always exceeded that of DWI except for specificity (Table 5, Fig. 6).

## Discussion

In univariate analysis, the multiparametric MRI-based genetic risk prediction with the conventional anatomical whole-body MRI, spinal diffusion-weighted MRI, and spinal dynamic contrast-enhanced MRI protocol identified eight significant features but none of them showed significance after statistical correction, making individual feature selection moot. As can be observed in Table 2, an abundance of (combinations of) CAs can be present in MM patients in different stages of the disease. Each of these specific CAs has consequences for the physiology and metabolism of the MM cells. As such, a complex interplay exists between CAs and physiological changes in the BM. In this way, different effects of the CAs on both the anatomical and functional MRI sequences occur at the same time, making that multiple features change simultaneously (features on BM composition, neovascularization, capillary permeability, bulk water flow, interstitial composition, cell density …), reducing the discriminative power of individual features to assess the cytogenetic risk.

Thus, model-based selection of a combination of features was performed to identify a multiparametric MRI signature to predict the cytogenetic risk. Different models were built and tested including models using all features and models using only the top three most predictive MRI features (Gillies’ rule to reduce overfitting and to increase generalizability of study results to other patient cohorts instructs that only one parameter or feature can be included for every 10 study patients). The multiparametric MRI top three features model performed best in predicting high-risk MM with a ROC-AUC 0.80, sensitivity 0.70, and specificity 0.81. The top three features model performed better than the all-features models including all 303 initially identified MRI features. This can be explained by the fact that the majority of identified MRI features were not meaningful to predict the cytogenetic risk and only introduced noise in the models, reducing the statistical performance. The conventional anatomical whole-body MRI top three models performed better than the spinal DCE-MRI or spinal DWI model separately. Furthermore, the performance of the top three spinal DCE-MRI model always exceeded that of spinal DWI except for specificity. These results highlighted the increased predictive performance of the multiparametric MRI model against the conventional anatomical whole-body MRI model alone. However, the conventional anatomical whole-body MRI model alone proved its merit against spinal DCE-MRI and spinal DWI models alone.

Previous studies investigated the potential of MRI to predict cytogenetic risk status in MM patients on specific MRI sequences and with various techniques. None of them assessed the potential of extensive qualitative/(semi-)quantitative whole-body multiparametric MRI as used in the current study. Jianfang et al. have built a spinal T1-/FST2-weighted model where the two-sequence model yielded the best performance (ROC-AUC 0.82/sensitivity 0.84/specificity 0.68) in the validation cohort [70]. Their preliminary study provided a T1-/T2-/FST2-weighted MRI model, based on a larger study sample and showed a slightly different performance (ROC-AUC 0.86/sensitivity 0.79/specificity 0.79/accuracy 0.79 in the validation cohort) [18]. Similar statistical metrics are obtained in our study. In comparison, our model is less sensitive (0.84/0.79 vs. 0.70), but more specific (0.68/0.79 vs. 0.81). Although both studies have similar distribution of intermediate-/high-risk and standard-risk cytogenetics, similar high-risk CA definition and similar region-of-interest segmentation methods, differences are the absence of second and high-order feature analysis and the addition of DWI/DCE-MRI sequences in the current study. Regarding infiltration patterns, Koutoulidis/Moulopoulos/Basiouny et al. demonstrated that a diffuse infiltration pattern was associated with high-risk CAs, increased BM microvascular density, elevated serum lactate dehydrogenase, anemia, worse response to conventional chemotherapy, and a worse prognosis. Diffuse pattern along with ISS stadium III and high-risk CAs identified a very high-risk group with poor median survival (21 months) and only a 35% 3-year OS [71–74]. In our study, BM infiltration pattern was not recognized as a good discriminator between cytogenetic risk groups, possibly because a multi-label classification of infiltration pattern was performed (four possible labels: focal, salt-and-pepper, diffuse, focal combined with diffuse or salt-and-pepper), which reduced the discriminative power of each label category in this smaller cohort study. Moreover, a focal pattern was only present in the high-risk cytogenetic group. Walker et al. demonstrated that the presence of > 7 focal MRI lesions is an independent survival predictor and is associated with elevated lactate dehydrogenase, C-reactive protein, and creatinine levels, and decreased albumin levels. However, it is not associated with the presence of high-risk CAs or with the β2-microglobulin level [75]. In our study, more (high-risk 85% versus standard-risk 56%) and larger (high-risk 57 mm^3^ versus standard-risk 10 mm^3^) focal lesions ≥ 5 mm were present in the intermediate-/high-risk group. Regarding DCE-MRI, Hillengass et al. demonstrated that high-risk CAs are significantly correlated with at least one DCE-MRI finding (aberrant “focal” microcirculation pattern, increased Amplitude A/Kep) and concluded that these high-risk CAs trigger the angiogenic switch [76]. In our study, no significant vascularization pattern differences (steep versus slow TIC) were identified between different cytogenetic groups. However, it should be noted that both groups tended to have a steep time-intensity curve which can help in the discrimination of high-risk against low-risk MM precursor states. Regarding DWI, Reem et al. demonstrated that ADC values < 770 × 10^-6^mm^2^/s correlated with diffuse BM infiltration which was indirectly related to high-risk CAs. A focal pattern on the contrary correlated with higher ADC values of 1046 × 10^-6^mm^2^/s [74]. In the current study, no significant difference was demonstrated.

Regarding limitations, retrospectively only a small patient cohort could be identified which was untreated, underwent the complete extensive MRI protocol correctly, and had a BM biopsy within three months from MRI. Moreover, patients presenting with alarming situations and symptoms with full-blown MM often undergo direct treatment before the MRI examination in clinical routine. As such, these patients were not included in this study. On the other hand, a broad range of real-world data regarding newly diagnosed untreated MM patients was included. In this way, this study can be seen as a representation of the real-world situation, encountered in a day-to-day clinical practice of a radiology department. Using real-world data, and not rigid study designs, offers the ability to assess the generalizability of methods (in this case of the statistical models to predict cytogenetic risk in newly diagnosed untreated MM) to clinical practice. In addition, only 21/31 patients presented with focal lesions, resulting in a restricted cohort for comparing focal lesions between the cytogenetic risk groups. More and larger prospective studies are required to assess generalizability to other cohorts. Second, (semi-)quantitative DCE-MRI and DWI features are easy to calculate and robust but are sensitive to variations between MRI protocols, for which external validation is necessary [55, 57]. This was not performed, as no dataset with identical scan protocol was available. To compensate, internal cross-validation and testing was performed. Limited data availability and imaging protocol variation is a concern in multiparametric studies, and larger heterogeneous multicenter studies with identical standardized scan protocols and external validation are required. This will help to reduce propagation of error through a feature extraction pipeline, avoid over- and underfitting, and improve the robustness and generalizability. The Quantitative Imaging Biomarker Alliance (QIBA) standardizes imaging protocols to ensure inter- and intra-machine reproducibility [77]. In MM, the Myeloma Response Assessment and Diagnosis System (MY-RADS) has been introduced to specifically address this issue [78]. Although the fact that these guidelines were already published in 2019, a large variability in clinically used MRI protocols still exists. By technically and clinically assessing an extensive conventional anatomical and functional MRI protocol in the current study, a direct comparison of the diagnostic performance in cytogenetic risk prediction of different scanning protocols including or excluding certain sequences can be performed (comparing it to an increased or decreased scanning time). A third limitation was not using second- and high-order statistical features. Nevertheless, good model performance was achieved by using SIRs and by adding spinal DWI and spinal DCE-MRI to the protocol. Presumably, high-order features could further positively influence the model’s sensitivity and statistical performance, considering the radiomics signature of Jianfang et al. A fourth limitation was due to the intra-tumoral and intra-patient spatial genomic heterogeneity at the chromosomal and mutational level in MM due to secondary acquired CAs [13]. This was reflected as samples collected in focal lesions, spine, and iliac crest differ genetically, confounding statistical results [79–81]. Contrarily, initiating disease driving events such as IgH translocations and hyperdiploidy was shared among different sites [13, 77, 82]. As such, high-risk CAs can be restricted to one site and absent at the iliac crest [70, 82]. Part of future prospective studies should be a multi-region imaging-guided biopsy and genetic analysis strategy with point-to-point comparison of cytogenetic risk and imaging features.

In conclusion, this multiparametric MRI signature opens opportunities and provides both clinical and technical insights for further noninvasive genetic risk stratification in newly diagnosed MM patients, overcoming sampling bias.

### Supplementary Information

**Supplementary Material 1.**

## Data Availability

Data, including clinical information and medical imaging, can be obtained from the data provider (Ghent University Hospital, B-9000-Ghent, Belgium), upon request and upon approval of the local Institutional Review Board.

## Abbreviations

(FS)T2: (Fat-saturated) T2-weighted MRI
ADC(R): Apparent diffusion coefficient (ratio)
AT(R): Arrival time (ratio)
b0(R): B0 diffusion sensitizing gradient (ratio)
b1000(R): B1000 diffusion sensitizing gradient (ratio)
BM: Bone marrow
bslope(R): Diffusion sensitizing gradient slope (ratio)
*b*-value: Diffusion sensitizing gradient
CA: Cytogenetic abnormality
CSF: Cerebrospinal fluid
DCE-MRI: Dynamic contrast-enhanced MRI
del: Deletion
DWI: Diffusion-weighted imaging
iAUC-TCC(R): Initial area-under-the-curve of the time-concentration curve (ratio)
iAUC-TIC(R): Initial area-under-the-curve of the time-intensity curve (ratio)
iFISH: Interphase fluorescence in-situ hybridization
IgH: Immunoglobulin heavy chain
IMWG: International myeloma working group
Kep(R): Rate constant (ratio)
Ktrans(R): Volume transfer constant (ratio)
L3: Third lumbar vertebra
LASSO: Logistic least absolute shrinkage and selection operator
MM: Multiple myeloma
M-protein: Monoclonal protein
mSMART: (Updated) Mayo Clinic risk stratification for multiple myeloma
*n*: Number
NPV: Negative predictive value
OS: Overall survival
PEI(R): Positive enhancement integral (ratio)
PPV: Positive predictive value
PR-AUC: Precision-recall area-under-the-curve
R2-ISS: Second revision of the international staging system
ROC-AUC: Receiver operating characteristic area-under-the-curve
SI(R): Signal intensity (ratio)
T1: T1-weighted MRI
T10: Tenth thoracic vertebra
TCC: Time-concentration curve
TIC: Time-intensity curve
TTP(R): Time-to-peak (ratio)
Ve(R): Extracellular extravascular space volume (ratio)
