# Supplementary material for: Predicting cytogenetic risk in multiple myeloma using conventional whole-body MRI, spinal dynamic contrast-enhanced MRI, and spinal diffusion-weighted imaging
Source: Insights Imaging. 2024 Apr 10;15:106. doi: 10.1186/s13244-024-01672-1 (PMC11006637; doi:10.1186/s13244-024-01672-1)
Supplement: Supplementary file 1 — Supplementary Material 1. [file 13244_2024_1672_MOESM1_ESM.pdf]

Predicting cytogenetic risk in multiple myeloma using conventional whole-body MRI, spinal dynamic contrast-enhanced MRI, and spinal diffusion-weighted imaging

ELECTRONIC SUPPLEMENTARY MATERIAL

Statistical analysis and modeling – extended version

To find imaging features or combinations of imaging features that are discriminative of genetic risk profile, both univariate and model-based methods were used. For the univariate analyses, Wilcoxon rank sum tests were performed. Missing values were excluded for these analyses and an adjustment for multiplicity was performed through application of the procedure of Benjamini and Hochberg, so as to control the false discovery rate at 5%[1]. For the model-based analyses, a pipeline was set up, consisting of preprocessing, feature selection, model building and model selection.

The preprocessing consisted of centering and scaling each numeric feature by subtracting the median and dividing it by its interquartile range. For each feature containing missing values, a missing value indicator feature was created, after which numeric missing values were imputed by the median and categorical missing values were imputed by the mode. Categorical features were then recoded into dummy features through one-hot encoding.

The feature selection consisted of several steps. In a first step, non-dummy features with both a frequency ratio (i.e. the frequency of the most prevalent value divided by the frequency of the second most prevalent value) higher than 19 and a unique values ratio (i.e. the number of unique values divided by the total number of values) lower than 0.1 were excluded. In a second step, a random forest was trained containing 500 trees and using the truncated square root of the number of input features as the number of randomly selected features in each tree. Based on the variable importance, a ranking of the features could then be performed after which a percentage of best performing features was selected.

Because imbalances between the most and least prevalent target class is known to be disadvantageous for the prediction model performance, we applied ADASYN (ADAptive SYNthetic sampling for imbalanced learning)[2]. ADASYN iteratively draws

a random minority sample, giving samples closer to the majority class a larger probability of being drawn, after which a new data point is randomly created on the line between the selected sample and one of its k (here equal to five) nearest minority neighbors. This process is repeated until the number of observations is equal in both classes.

Finally, as a last preprocessing step, an intercept column was added.

With this preprocessing pipeline, different linear (LASSO - logistic least absolute shrinkage and selection operator) and nonlinear (random forests, radial basis function kernel support vector machines and neural networks) classification methods without extensive hyperparameter tuning were preliminary explored. This preliminary exploration showed similar performances for the linear and nonlinear methods and thus motivated the choice for the logistic LASSO.

The complete pipeline contained two tunable hyperparameters: percentage of features to select in the random forest feature selection step and the LASSO penalty parameter. Both hyperparameters were optimized simultaneously with Bayesian optimization. Bayesian optimization fits a surrogate model that maps the tuning parameters to the expected performance (here receiver operating characteristic area-under-the-curve) and uses this surrogate model to draw new candidate hyperparameters that look promising. To start the optimization, an initial random sample of five hyperparameters was drawn and the optimization stopped after 50 iterations or after 10 iterations without improvement.

Because of the small sample size, a train-test split of the data was not possible, as the test data would then be too small to draw meaningful conclusions. Instead, a 25-times repeated k-fold cross-validation to estimate the performance (accuracy, F1, precision-recall area-under-the-curve, precision, recall, receiver operating characteristic area-under-the-curve, negative predictive value and specificity) and bootstrapping (B=25) nested within each fold to cross-validate the hyperparameter tuning was performed. k=4 was chosen as it offered a balance in training and test set size.

The performance of four different statistical models was tested including (1) all features of the multiparametric MRI examination (conventional anatomical whole-body MRI + spinal dynamic contrast-enhanced MRI + spinal diffusion-weighted

imaging), (2) all conventional anatomical whole-body MRI features only, (3) all spinal dynamic contrast-enhanced MRI features only and (4) all spinal diffusion-weighted imaging features only. As a final step, four final models were tested with the three most frequently LASSO-selected features to test the best performing models in each group. For this, Gillies' rule of thumb (one included parameter for every 10 study patients to avoid overfitting) was applied.

All analyses were performed with R version 4.2.2 (Microsoft Corporation) on a Windows 64-bit machine, using the tidymodels, recipeselectors and themis packages[3-6].

Session info

R version 4.2.2 (2022-10-31 ucrt) (Microsoft Corporation)

Platform: x86_64-w64-mingw32/x64 (64-bit) (Microsoft Corporation)

Running under: Windows 10 x64 (build 19044) (Microsoft Corporation)

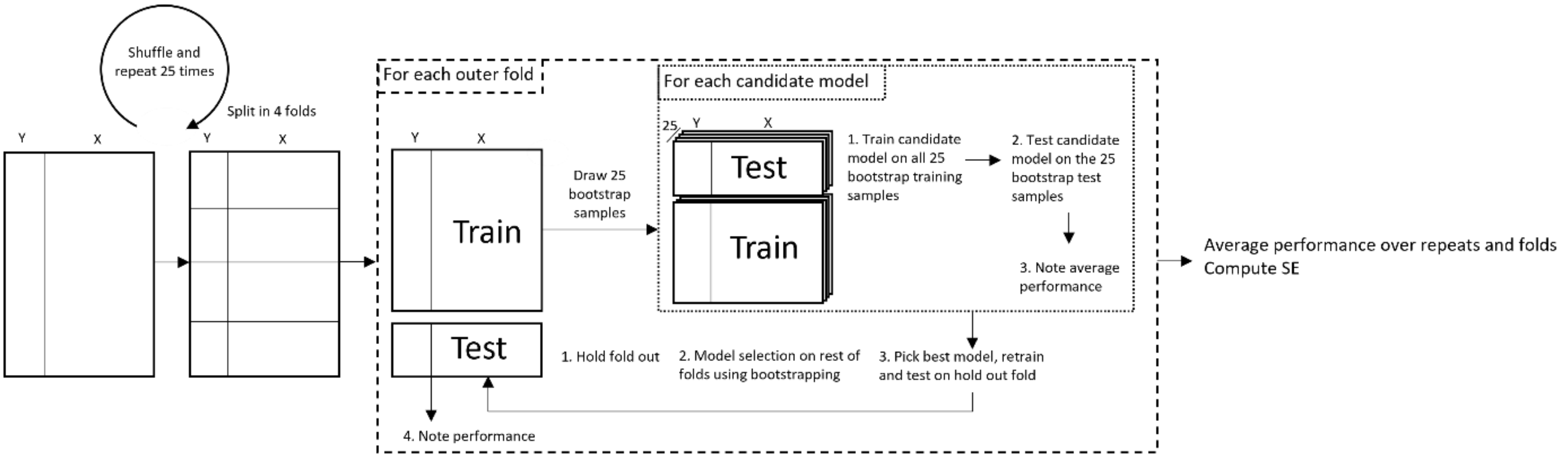

Supplementary Fig. 1 Nested cross-validation scheme for feature and model selection (inner cross-validation) and performance estimation (outer cross-validation). The entire patient population is split in a training and independent test set for each fold, ensuring equal class distribution within folds. The training set is used to select features, train candidate predictive models and validate their performance internally. Next, the best candidate model is chosen, retrained, and tested on the independent test set. After all repetitions, the average performance is calculated together with the standard error. SE=standard error.

References

1. Benjamini Y, Hochberg Y (1995) Controlling the False Discovery Rate: a Practical and Powerful Approach to Multiple Testing. J R Stat Soc. DOI: 10.2307/2346101

2. Haibo H, Yang B, Garcia EA, Shutao L (2008) ADASYN: Adaptive synthetic sampling approach for imbalanced learning. 2008 IEEE International Joint Conference on Neural Networks (IEEE World Congress on Computational Intelligence). IEEE. DOI: 10.1109/IJCNN.2008.4633969

3. R: A language and Environment for Statistical Computing, R Core Team and R Foundation for Statistical Computing (2022) Available via https://R-project.org/. Accessed 24 July 2023.

4. Tidymodels: A collection of packages for modeling and machine learning using tidyverse principles, Max Kuhn and Hadley Wickham (2020) Available via https://www.tidymodels.org. Accessed 24 July 2023.

5. Recipeselectors: Additional recipes for supervised feature selection to be used with the tidymodels recipes package, Steven Pawley (2022) Available via https://github.com/stevenpawley/recipeselectors. Accessed 24 July 2023.

6. Themis: Extra Recipes Steps for Dealing with Unbalanced Data, R Package Version 1.0.0 (Emil Hvitfeldt) (2022) Available via https://CRAN.R-project.org/package=themis. Accessed 24 July 2023.
